# Rosehip (*Rosa canina* L.) Meal as a Natural Antioxidant on Lipid and Protein Quality and Shelf-Life of Polyunsaturated Fatty Acids Enriched Eggs

**DOI:** 10.3390/antiox11101948

**Published:** 2022-09-29

**Authors:** Petru Alexandru Vlaicu, Arabela Elena Untea, Raluca Paula Turcu, Tatiana Dumitra Panaite, Mihaela Saracila

**Affiliations:** Feed and Food Quality Department, National Research-Development Institute for Animal Biology and Nutrition, 077015 Balotesti, Romania

**Keywords:** egg quality, fatty acids, amino acids, shelf-life, antioxidants, lipids, proteins, rosehip meal

## Abstract

Eggs are a common food of animal origin, inexpensive, and rich in bioactive substances with high biological value. Eggs enriched in polyunsaturated fatty acids (PUFA) are extremely desired by the progressive consumer. However, during storage, eggs undergo some physiochemical changes, which decrease their value. In this regard, the effect of dietary rosehip meal and flaxseed meal on hens’ egg quality characteristics, amino acids, fatty acids, health-related indices, antioxidant capacity, total polyphenols content, and shelf life was examined. For this study 120 Tetra SL laying hens, 29 weeks of age, were fed, for 4 weeks, three diets that included control (basal diet—RF0), basal diet + 1.5% rosehip and 7% flaxseed meal (RF1), and basal diet + 3% rosehip and 7% flaxseed meal (RF2). Productive performance of hens were recorded. The content of essential amino acids (EAA), antioxidant amino acids (AAA), and sulfur amino acids (SAA) was higher in RF1 and RF2, compared with RF0. Eggs belonging to the RF1 and RF2 groups had significantly (*p* < 0.05) higher content of n-3 PUFAs, especially linolenic and docosahexaenoic acids. Total antioxidant capacity and polyphenol content increased in both rosehip supplemented groups, but especially in RF2. Moreover, eggs from RF1 and RF2 groups maintained significantly higher egg quality parameters after storage for 14 and 28 days in the refrigerator (5 °C) and ambient temperature (21 °C), compared with those from the RF0 group. In the Haugh unit, yolk and albumen pH presented better values in RF1 and RF2 eggs compared to the RF0 eggs.

## 1. Introduction

Eggs are food products of animal origin consumed and served in a large variety of ways all over the world, like no other animal origin product. For this reason, due to consumers’ demand in the last decades, eggs have become not only a day-to-day consumed food but also a worldwide phenomenon to be used as a functional food [1]. Producing eggs that have the value of functional food is a highly investigated subject and receives substantial attention because this aspect includes both enhanced nutritional quality parameters, as well as their shelf-life. Some studies from written questionnaires indicate that most consumers reported that freshness of food, health enhancement and functionality to prevent some diseases are the major criteria when selecting foods [2,3]. Numerous works have investigated the multiple ways to enhance or modify the composition of eggs by using supplements with different nutrients or biologically active compounds like vitamins, minerals, carotenoids, and fatty acids. Because egg composition can be modified by manipulating laying hens’ feed, many of these studies succeeded and obtained significant results, especially those with enrichment in n-3 polyunsaturated fatty acids (n-3 PUFA). Several reports concluded that flaxseed carries functional ingredients like proteins, amino acids [4], dietary fibers [5] and is a significant source of n-3 PUFA [6]. All these nutrients provide health benefits, preventing serious diseases like obesity, cancer, coronary disease, and bone and renal disorders [7]. However, it was reported that this enrichment increases the susceptibility to lipid and protein oxidation, decreases shelf-life or gives undesirable odors in eggs which represents a major problem for consumers and producers [6]. To avoid such unwanted effects, a natural way to extend the intrinsic antioxidant concentration in eggs and maintain their shelf-life is by using another natural source as an antioxidant, together with flaxseed meal in laying hens’ diets. One such unexploited nutritious candidate is rosehip (*Rosa canina* L.) meal. In some cultures, rosehip has been used as a medicinal remedy for over 2000 years, but the actual consumption of this fruit is very popular in European countries like Romania, Poland, Portugal, Germany, Finland, and Sweden [8]. Rosehip is very rich in vitamins (A, B, C, D, and E), minerals (Ca, Fe, K, Mg, Mn, S, Si, and Se), polyphenols, and has high antioxidant capacity [9,10,11], with a moderate level of amino acids and fatty acids. Currently, rosehip fruits are used in the fruit processing industry for different purposes [8]. However, as in the case of other products, processing waste management is an important issue, large quantities of by-products with a valuable content of bioactive compounds with health-promoting properties being generated. This is the case with rosehip meal, which only recently gained attention, as the need for healthy foods escalated. Nutritional and chemical analyses have suggested the usage of rosehip meal might be a good candidate to obtain eggs with functional properties [12]. To date, there are only a few studies in which rosehip was tested as a functional ingredient in hens’ diets, [13,14] and to our knowledge, there are no studies in which rosehip in flaxseed meals were used together to obtain a potential functional food product, with improved nutritional qualities, beneficial for consumers. 

Therefore, this study aimed to evaluate the effects of rosehip meal as the natural antioxidant source on nutritional qualities, amino acids, fatty acids, health-related indices, antioxidant capacity, total polyphenols content, shelf-life, polyunsaturated fatty acids in eggs enriched by using flaxseed meal. 

## 2. Materials and Methods

### 2.1. Ethical Procedure

All procedures used in the current study were approved by the Ethics Committee of our institution, according to an experimental protocol, and complied with Law 43/11.04.2014, Directive 2010/63/EU, regarding the use of animals for experimental purposes.

### 2.2. Experimental Materials

The rosehip and flaxseed meals were purchased from a company (S.C. 2-eProd S.R.L.), located in Teleorman County, Romania (RO). The dietary ingredients used in this study were obtained by cold pressing technology after the oil extraction process from rosehip and flaxseed. 

### 2.3. Birds’ Husbandry

The experiment was conducted for five weeks on 120 Tetra SL laying hens, between 29 and 33 weeks of age, randomly distributed into three equal groups, each having 40 laying hens (10 repetitions of 4 hens per pen). The hens were raised in an experimental hall equipped with Zucami three-tier metallic digestibility cages (60 cm width × 60 cm length × 40 cm height), which allowed for recording daily feed intake and egg production. Minimum and maximum temperatures and humidity were monitored daily with a Big Dutchman automatic controlled microclimate system (temperature: 20 to 22 °C, humidity 56 to 62%). According to the management breeding guide of the hybrid, the animals were subjected to a constant photoperiod of 16 h light and 8 h dark.

### 2.4. Experimental Diets

Diets were formulated based on the chemical analysis results of the ingredients used, and supplied the layers’ nutritional requirements, considering the nutritional requirements of the hens. The treatments consisted of one basal diet (RF0) containing as major ingredients corn, soybean meal, and corn gluten. The two experimental diets were formulated with the addition of 1.5% rosehip meal with 7% flaxseed meal and (RF1) and 3% rosehip meal with 7% flaxseed meal with (RF2). All diets were equally iso-protein and iso-caloric having 17.50% crude proteins and 2780 metabolizable energy (kcal/kg). The feed and water were provided ad libitum. Table 1 shows the ingredients of dietary treatments.

### 2.5. Laying Performance and Egg Production 

Daily feed intake (DFI, g/bird per day), feed conversion ratio (FCR, kg of feed/kg of eggs), egg production (%), and egg weight (g) were monitored and recorded daily. Egg mass was calculated by multiplying the laying rate (%) by the average weight of eggs (g) divided by 100. 

### 2.6. Egg Sampling 

Egg quality was measured on 20 eggs from each treatment (*n* = 60) collected at the end of the trial and submitted to the quality analysis to represent the characteristics of fresh eggs on the last experimental day. After these measurements were done on fresh eggs, the yolk samples were pooled to determine amino acids (AA), fatty acids (FA), total antioxidant capacity (TAC) in the yolk, and albumen, and total polyphenol content (TPC) in the egg. To determine the effect of dietary treatments on shelf-life 40 eggs/group (120 eggs in total) with similar weight were marked carefully before storage according to the date of production and placed in paper egg trays. Further, half of the eggs (*n* = 60) were stored under ambient conditions at 21 °C and the other half (*n* = 60) were stored in the refrigerator at 5 °C. After 14 and 28 days of storage time respectively, 10 eggs/ group, were sent to the laboratory for egg quality analyses, both from eggs stored in the refrigerator and from those stored at ambient temperature.

### 2.7. Primary Chemical Analysis

The nutritional composition analyses from the rosehip and flaxseed meals, compound feeds and eggs were determined on samples dried at 65 °C, by using standardized methods performed according to Regulation (CE) no 152/ 2009. SR EN ISO 5983-2:2009 for crude protein (Kjeltec auto 1030; Tecator Instruments, Hoganas, Sweden, SE); SR EN ISO 6492:2001 for crude fat (Soxtec 2055; Foss Tecator, Hoganas, Sweden, SE); and SR EN ISO 6865:2002 for crude fiber (Fibertec 2010 System; Foss Tecator, Sweden, SE), as described elsewhere [15].

### 2.8. Amino Acids Analyses

#### 2.8.1. Amino Acid Content (AA)

The amino acid content of egg yolk samples was performed using a reversed-phase high-performance liquid chromatography (RP-HPLC) method on a HyperSil BDS C18 column, with silica gel, dimensions 250 × 4.6 mm, particle size 5 μm (Thermo-Electron Corporation, Waltham, MA, USA), according to the method described by [16].

#### 2.8.2. Estimations of Egg Yolk Protein Quality Indices

The quality of protein given by the AA content was determined by estimating the ratio of different AA determined in the egg yolk samples as recommended by the WHO/FAO/UNU [17]. The essential amino acids (EAA) to total amino acids (TAA) ratio; the cysteine to sulfur amino acids (SAA) ratio; the EAA to non-essential amino acids (NEAA); the total of the aromatic amino acids (TAAA) and antioxidant amino acids (AAA) were also calculated. 

### 2.9. Egg Yolk Fatty Acids Analyses

#### 2.9.1. Fatty Acids Content 

The fatty acid (FA) analyses from samples dried at 65 °C were determined using fatty acid methyl ester (FAME) gas chromatography as previously presented [18]. We used a Perkin Elmer-Clarus 500 (Waltham, MA, USA) chromatograph equipped with a flame ionization detector (FID) and fitted with a BPX70 capillary column (60 m × 0.25 mm i.d., 0.25 μm film thickness). FAME identification was done by comparison with retention times of the known standards. The average amount of each FA was used to calculate the sum of the total saturated (SFA), unsaturated (UFA), monounsaturated (MUFA), and polyunsaturated (PUFA) fatty acids. 

#### 2.9.2. Estimation of Lipid Health-Related Quality Indices

All the health-related quality indices considered to be estimated in this study have, specific only for eggs, been classified into categories based on the aim for which they were conceived, and were separated into three categories: nutritional, qualitative, and metabolic indices. We used appropriate formulas for each estimated lipid quality indices, as they were validated previously in different reports [6,19,20].

### 2.10. Total Polyphenol Concentration and Antioxidant Capacity Analyses 

The total polyphenol concentration (TPC) was determined spectrophotometrically in the methanolic extract samples, using a UV–Vis Thermo Scientific spectrophotometer (Cambridge, UK) while the total antioxidant capacity (TAC) of the methanol extracts was determined using the DPPH method, with a UV–Vis Analytik Jena Specord 250 Plus spectrophotometer (Cambridge, UK) with the thermostatic carousel as presented elsewhere [21]. 

### 2.11. Shelf-Life of the Eggs

The egg quality parameters that influence the shelf-life of the eggs: egg weight (g), albumen weight (g), yolk weight (g), shell weight (g), eggshell thickness (mm), eggshell breaking strength (kf), albumen pH, yolk pH, yolk color and Haugh units, from fresh and stored eggs were determined as previously presented [22]. 

### 2.12. Statistical Analysis 

The statistical model applied used one-way analysis of variance (ANOVA), using StatView for Windows (SAS, version 6.0, BrainPower Inc., 24009 Ventura Blvd. Suite 250, Calabasas, CA 91302, USA). Significance between individual means was identified using the Fisher test. When the one-way ANOVA results indicated *p*  <  0.05, the means were considered significantly different. The 3 means were compared two by two, using the Fisher test, and the significant differences (*p*  <  0.05) were marked in the tables by superscripts (a, b, c). For estimating how close the individual sample mean is to the system mean, the standard error of the mean (SEM) was calculated. The Pearson correlation for amino acids and fatty acids, and Principal Component Analysis (PCA) were obtained from the corresponding function of the Matlab & Simulink (version 2020, MathWorks Inc Bartok B. ut 15/d 1114 Budapest, Hungary) software package, used to reveal the correlation structure between the investigated parameters. 

## 3. Results

### 3.1. Chemical Composition of the Rosehip and Flaxseed Meal

Flaxseed meal showed a higher concentration of crude protein and crude fat, while rosehip meal showed a higher concentration of crude fiber, which limits its usage in laying hens’ diets (Table 2). The antioxidant compounds were determined only in rosehip meals. The fatty acid (FA) profile of rosehip meal showed higher concentrations of oleic and linoleic FA, whereas palmitic, stearic, and α-linolenic acids were higher in flaxseed meal. The amount of total essential amino acids (EAA) and non-essential amino acids (NEAA) was lower in the rosehip meal than in the flaxseed meal.

### 3.2. Effect of Rosehip and Flaxseed Meals on Laying Hens’ Performances

The effects of dietary treatments showed significantly lower daily feed intake in hens fed RF1 and RF2 diets compared with those fed the RF0 diet. No significant effect was found for feed conversion ratio. Laying percentage and egg mass, significantly increased in the RF2 group, compared with the RF0 group, while RF1 was higher than RF0 but without significant effect (Table 3). 

### 3.3. Effect of Rosehip and Flaxseed Meal on Amino Acid Profile in Egg Yolk

In this study, we detected 15 egg yolk AA from which eight were EAA and seven NEAA (Table 4). The contents of EAA were significantly higher in the RF1 and RF2 samples compared to the RF0 samples, while the NEAA was significantly higher in the RF0 samples compared with RF1 and RF2. From the EAA for human consumption, arginine, lysine, and threonine were present in higher concentrations, followed by isoleucine, leucine, and valine, in RF1 and RF2 samples compared with RF0. From the NEAA, variable concentrations were determined among the groups. Overall, this supplement has a significant potential to increase all EAA in the egg yolk protein, revealing significant effects of rosehip and flaxseed meals on the protein quality of the eggs. 

Table 5 shows the data for AA quality indices. The results indicate that protein egg yolk from RF1 and RF2 presented a higher (*p* < 0.05) concentration of AA with antioxidant potential (AAA), sulfur amino acids (SAA) and total aromatic amino acids (TAAA) compared with RF0. The ratios for aromatic (TAAA) to total (TAA), essential (EAA) to total (TAA) and essential to non-essential (NEAA) ratios were higher in RF1 and RF2 compared with RF0 egg yolk protein samples. However, the cystine to SAA was higher in the RF0 samples compared to RF1 and RF2. 

**Figure 1:**
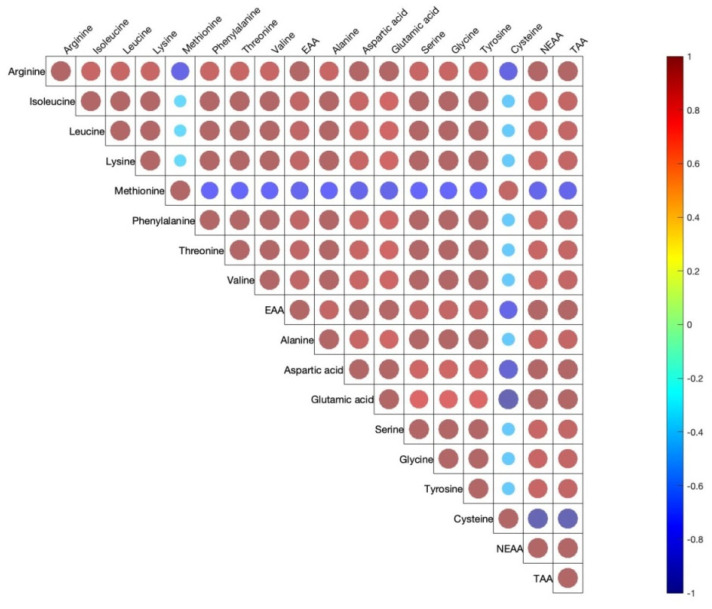
Pearson correlations among egg yolk amino acids. The correlations are expressed by circles. Red and blue circles indicate positive or negative correlations in each pair, respectively (*p* < 0.05). Shapes of circles are dependent on weak to strong correlations from big to small circles.

Figure 1 shows the correlation coefficients between the 15 amino acids determined in egg yolks. The EAA, NEAA, and TAA are also included. Across 15 yolk amino acids, there were moderate to high positive correlations with only a few negative correlations. All EAA and AAA were moderately or strongly positively correlated among them. 

### 3.4. Effect of Rosehip and Flaxseed Meal on Fatty Acid Content in Egg

The FA content of the eggs is shown in Table 6. From the total SFA, palmitic and stearic acids were the most abundant especially in the RF0 compared with RF1 eggs. Oleic acid was significantly higher in the RF0 eggs compared with RF1 and RF2 eggs, which led to significantly higher total MUFA. Linoleic acid was significantly higher in RF1 and RF2 eggs compared with RF0, while arachidonic acid was significant in RF0 eggs compared with RF1. No significant effect was noted in the total n-6, although the concentrations were higher in the RF0 eggs compared with RF1 and RF2. The most significant variation was observed for the total n-3. The linolenic (ALA) and docosahexaenoic (DHA) acids were almost five times higher in the eggs from RF1 and RF2 groups compared to the RF0 eggs. This effect led to a significant alteration in the total PUFA, in the favor of RF1 and RF2 eggs compared to the RF0 eggs. 

**Figure 2:**
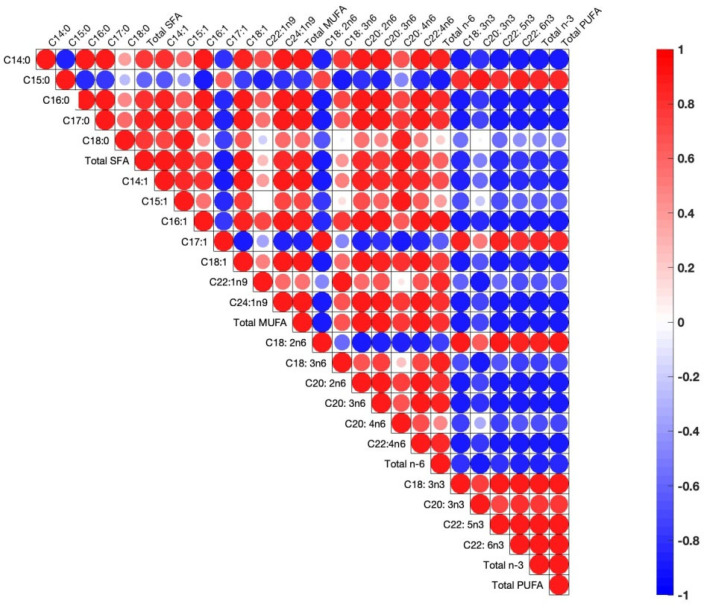
Pearson correlations among egg yolk fatty acids. The correlations are expressed by circles. Blue and red circles indicate positive and negative correlations in each pair, respectively (*p* < 0.05). Shapes of circles are dependent on weak to strong correlations from big to small circles.

During correlation analysis between fatty acid concentrations in the egg yolks, there are positive correlations between pairs of fatty acids, which implies a metabolic pathway whereby increases in one of the fatty acids engender increases in the other, or vice versa when the correlation is negative (Figure 2). 

The indices considered for estimation in this study, specifically only for eggs, have been separated into groups based on the aim for which they were conceived (qualitative, nutritional, and metabolic), as summarized in Table 7. The qualitative indices were all influenced among the groups, but the most significant alteration was observed between n-6/n-3 and linoleic/linolenic acids, both being significantly (*p* < 0.05) lower in RF1 and RF2 eggs compared to RF0. From the nutritional group, atherogenicity and thrombogenicity indices were significantly lower in RF1 and RF2 while the nutritive value, hypocholesterolemic/hypercholesterolemic and desirable fatty acids indices were significantly higher compared to RF0. Lastly, in the metabolic group, only the elongase and thioesterase indices were influenced, with no effect on Δ9 or Δ5/Δ6-desaturase. 

### 3.5. Effect of Rosehip Meal on TAC and TPC Determined in Eggs

**Figure 3:**
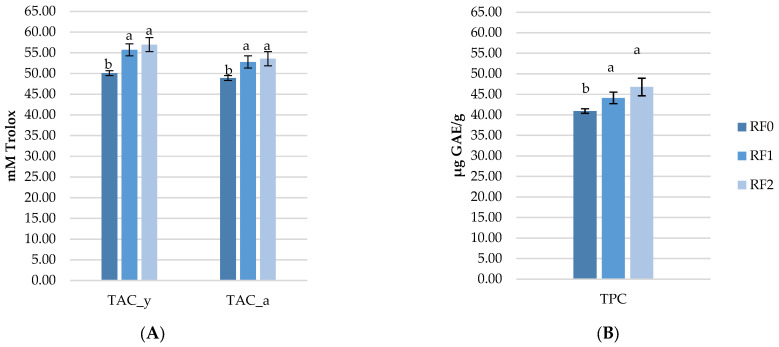
Effect of rosehip meal on the total antioxidant capacity determined in the egg yolk and albumen (**A**) and total polyphenols content (**B**) in polyunsaturated fatty acids enriched eggs. The significant differences (*p*  <  0.05) are marked in the tables by superscripts (a, b) after the 3 means were compared two by two, using the Fisher test. RF0: control diet; RF1: contains 1.5% rosehip meal and 7% flaxseed meal; RF2: contains 3% rosehip meal and 7% flaxseed meal.

Figure 3 presents the effect of rosehip meal on PUFA-enriched eggs. The total polyphenol content in egg (TPC) and total antioxidant capacity (TAC) determined in yolk and albumen (TAC_y and TAC_a), revealed that both RF1 and RF2 had significantly higher polyphenols and antioxidant capacity compared to the RF0 eggs. However, the RF2 had slightly higher concentrations of both TAC and TPC, due to a higher level of rosehip addition, but without significance compared to the RF1 eggs. 

### 3.6. Effect of Rosehip and Flaxseed Meal on Shelf-Life of Eggs

The results regarding the effect of rosehip and flaxseed meals on egg quality traits and shelf-life during 28 days of storage time at 5 °C and 21 °C are presented in Table 8. Storage time had a significant (*p* < 0.05) effect on freshness parameters (yolk pH, albumen pH, Haugh Units) and egg components. The temperature had a significant effect on all external and internal quality parameters, except for shell weight and shell quality (*p* > 0.05). The diet had a significant effect on freshness parameters (Haugh units, yolk pH, and albumen pH). The interaction between time x temperature was without any effect, as well as the interaction between time x temperature x diet. However, time x diet interaction had a significant effect on lipid parameters (yolk pH), while the interaction between temperature x diet had a significant effect on protein parameters (albumen pH). 

### 3.7. Principal Component Analysis (PCA)

**Figure 4:**
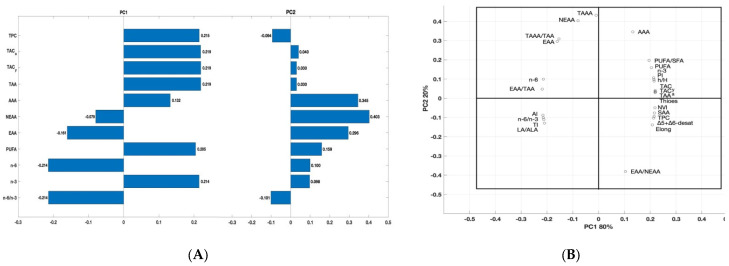
Results of the principal component analysis (PCA) for selected parameters of the egg yolks obtained from hens in the RF0, RF1, and RF2 groups, determined at the end of the study. (**A**) Partial sums of correlation between total polyphenol content (TPC), total antioxidant capacity in albumen (TAC_a) and yolk (TAC_y), sums of PUFA, n-3, n-6, n-6/ n-3 ratio, total amino acids (TAA), antioxidant amino acids (AAA), non-essential amino acids (NEEA) and essential amino acids (EAA) of the egg yolks obtained from hens in the RF0, RF1 and RF2 groups, determined at the end of the study. (**B**) PCA bi-plot (loading plot) for the PC1 and PC2 components. PC1 covered 80% of the variance and PC2 covered 20% of the variance.

To compare the partial sums of fatty acids (PUFA, n-3, n-6, and n-6/n-3), sums of amino acids, health-related indices, total antioxidant capacity, and total polyphenol content in the egg yolks obtained from hens in the RF0 versus RF1 and RF2 and to analyze the variability in their analysis’s variables, a principal component analysis (PCA) was applied (Figure 4). The first two principal components (PC), showed eigenvalues greater than or close to 1 (3.66 and 1.41 for PC1 and PC2), explaining 100% of the data variation. 

The first principal component PC1 (eigenvalue: 3.66) explained most of the variability of the data (80%) and was significantly negatively associated with NEEA, EAA, n-6, and n-6/n-3 (correlation −0.0079, −0.191, −0.871, and −0.871, respectively) and positively correlated with TAC_y, TAC_a, TPC, TAA, AAA, PUFA and n-3 (Figure 4A). The second component PC2 (eigenvalue: 1.44) explained less of the variance (20%) and was only negatively correlated with TPC (correlation: −0.014) and n-6/n-3 (correlation: −0.101). As the eigenvalue of PC2 was above 1, meeting Kaiser’s criterion of inclusion, a Cattell’s scree plot was performed to confirm that the usage of PC2 was appropriate. PCA loading plot for the first two components, which combined explain 100% of the variation of the data, showed that there was significant variability among calculated protein and lipid health-related indices (Figure 4B).

## 4. Discussion

### 4.1. Chemical Composition of the Rosehip and Flaxseed Meals

The nutritional composition, antioxidant compounds, fatty acid profile, and amino acid content in rosehip and flaxseed meal highlighted that both supplements have variable concentrations of these nutrients. Flaxseed meal presented high concentrations of crude protein and crude fat, while rosehip was noted to have a high concentration of crude fiber, which limits its usage in poultry diets. As it is well known, flaxseed meal proved to have a high concentration of n-3 PUFA, especially ALA, being more than three times higher than that determined in rosehip meal. The lowest ratio of n-6 to n-3 was also determined in flaxseed meal. In terms of AA content, flaxseed meal was dominant for the EAA especially lysine, methionine, phenylalanine, and threonine, mostly due to its higher protein content. However, rosehip meal was added as a natural source of antioxidants and it was expected to be scarce in fat and protein content, but rich in polyphenols and antioxidant capacity. Previous investigations reported that the chemical composition determined in supplements such as flaxseed and rosehip meal differ due to their oil extraction method [23], harvest conditions and period [6], soil type [24], other climatic factors, or the cultivation country [25]. In a recent study on rosehip meal [26], harvested in Bulgaria, it was reported that the TAC was 9.436 mM Trolox, the TPC was only 16.52 mg GAE/g, and there were no flavonoids, which is very low compared with the chemical composition presented in the current study. This confirms that the above-mentioned factors have a significant effect on the chemical composition of the supplements. However, care must be taken because different chemical compositions of the supplements, will lead to different results when tested in animal feed.

### 4.2. Effect of Rosehip and Flaxseed Meals on Laying Hens’ Performances

Results from the performance of laying hens indicated that the addition of rosehip meal with flaxseed meal significantly reduced daily feed intake, but increased laying intensity and egg mass. Recently it was reported that 9% flaxseed meal affected feed intake and the feed conversion ratio [27], but with improved egg mass and laying intensity, which is similar to this study, while others reported no effect [28] or decreased feed intake [29]. With a focus on egg production and commercial farms, egg mass is a better index than laying intensity. In the present study, hens fed RF1 and RF2 diets had a higher value (*p* = 0.0302) of egg mass when compared to those fed RF0. Similar results were reported in different studies [6,27], revealing that flaxseed alone or in combination with antioxidant sources increased egg mass. In a different study [27], unprocessed 9% flaxseed meal without antioxidants, had a negative impact on laying hens’ performances. Similarly, Imran, [30], reported that 10% extruded flaxseed resulted in lower egg production and hens’ body weight. Another study reported that the effect of 0.5% rosehip meal alone had no effect on production performances [26], while others [31], reported that 10% to 15% addition resulted in increased feed consumption. These results might be caused by the poor digestibility of nutrients by the hens, showing negative or no effects on performances, which results in lower egg quality. Other factors that influence the performances are the age of the birds, strain, and the processing of meals. Unprocessed meals contain lots of anti-nutritional factors that have a negative impact on egg quality and animals’ performances. We noted that these inconsistencies in the reported results can come from the different experimental designs, different combinations, and concentrations of the supplements, age of the hen, and strain. 

### 4.3. Effect of Rosehip and Flaxseed Meal on Yolk Amino Acids and Protein Quality Indices

The nutritive value and functional properties of eggs make them an important animal protein source. The AAs are functional and structural units of protein, nutritionally classified into EAA and NEAA [32]. The EAA cannot be synthesized by the body while the NEAA is synthesized in the body. Both groups play vital physiological roles in the body after absorption, being assembled and metabolized to form proteins that are used to build different body tissues or to protect against some diseases [33]. Feeding the RF1 and RF2 diet to laying hens resulted in a significant (*p* = 0.0229) increase in the crude protein concentration in egg yolk compared with the RF0 group (Table 4). The present results showed that RF1 and RF2 samples had greater (*p* < 0.05) EAA content than RF0. Specifically, the same groups had a greater content (*p* < 0.05) of isoleucine, lysine, phenylalanine, and threonine than RF0. Cysteine and tyrosine from the NEAA group were also determined to be in higher concentrations in the RF1 and RF2 eggs compared to RF0. Although they are classified as NEEA in fact they are recognized as semi-essential AA because they can be synthesized from methionine and phenylalanine [34]. In terms of AA deposition into the eggs, chickens are capable of synthesizing de novo AA in a cell- and tissue-specific manner. Studies on AA retention and oxidation have shown that the efficiency of utilization is inherent to individual AA and may differ even under comparable dietary conditions [35], making it possible to obtain EAA-enriched eggs. Although in previous research studies, the albumen was considered the important source of AA, recently [36] showed that actually egg yolk provides the highest percentage of phenylalanine, methionine, lysine, isoleucine, valine, and threonine for an adult based on the RDA recommendations mentioned by the FAO [17]. 

From the estimated protein quality indices, the results indicate that RF0 had the lowest quality of protein in terms of TAAA and AAA (Table 5). The TAAA are related to food taste and are classified as umami, sweet, sour, bitter, salty, and astringent tastes. These entire groups of AA increase the taste quality of the product and consumer acceptance. For the AAA aspartic acid, lysine, methionine, and tyrosine were considered the main contributor to the antioxidant properties of eggs, from which RF2 had the highest cumulated value compared with RF0. Cysteine, methionine, tyrosine, and histidine were reported as being relatively easily oxidized [37], however, the antioxidant potential of rosehip meal, improves their antioxidant properties to prevent protein oxidation. The EAA/TAA ratio, cysteine/SAA ratio, as well as total SAA, were significantly higher in RF1 and RF2, compared with RF0 eggs, showing high variability for the determined AA. When comparing our results with previously published data on similar protein quality indices, we noted that some results are similar [36] or contradictory [38]. Although the diets and ingredients used in laying hens feed have significant implications on the protein quality deposited into the egg yolks. The strains or breed, environmental condition, storage time of the feed, poultry species, and egg part also led to different results. All in all, the nutritional value of AA in food mainly depends on the quantity and proportion of EAA. The World Health Organization (WHO) and Food and Agriculture Organization (FAO) 1973 recommended that the quantity of EAA in food should be considered an important criterion to evaluate the nutritional value of food protein. The lack of studies in which rosehip meal alone or in combination with flaxseed was tested on egg yolk protein quality makes it difficult to compare to our findings. 

The Pearson correlation showed that dietary supplements had moderate to high correlations among AA within each yolk. The correlation also showed that AA balance in the yolk can be regulated by the diets. Since egg yolk components already have some antioxidant properties, further investigations are needed to observe other correlations among egg yolk components in the future.

### 4.4. Effect of Rosehip and Flaxseed Meal on Yolk Fatty Acids and Lipid Quality Indices

The PUFAs are FA with important nutritional and physiological functions, some of them being essential to humans: linoleic acid (LA) from the n-6 group and α-linolenic (ALA), eicosapentaenoic (EPA), and docosahexaenoic (DHA) acids from the n-3 group. In laying hens, the egg yolk FA composition depends on liver lipid synthesis and the lipid components of the diet. Through manipulation of the diet, it is possible to modify the FA profile of the egg and increase its PUFA content. In the present study, the inclusion of 7% flaxseed meal rich in PUFA and 1.5 or 3% rosehip meal as antioxidant sources resulted in a significant increase of these beneficial FA in the egg yolk. The significant increase of ALA resulted in a significant conversion into DHA. Interestingly, the main n-3 PUFA deposited in the eggs lipids was ALA and DHA and not EPA (Table 6). However, EPA-enriched eggs are harmful to infants, due to their competition with arachidonic acid (C20:4 n-6) for incorporation into tissue phospholipids [39]. Another logical explanation is that due to elongation and desaturation, EPA was converted entirely into DHA in the hens’ livers, however, this process was not observed in chicken’s thigh meat, where EPA was determined [40]. A similar phenomenon was also observed by others when EPA was largely converted into DHA [41,42,43] when laying hens were fed flaxseed-based diets. The competition between n-6 and n-3 PUFA in the desaturation-elongation process was clearly in favor of n-3 PUFA. However, we observed that flaxseed is more efficient in increasing DHA and n-3 PUFA content in eggs compared with algae or microalgae [44]. In the study of Wu et al. [4], DHA content in n-3 PUFA enriched eggs and the total n-3 PUFA were lower compared to our data. Previously we observed that 9% flaxseed meal and 3% sea-buckthorn meal had a similar effect [6]. We can assume that the rosehip meal protected the lipid oxidation against reactive oxidative species, which resulted in higher n-3 PUFA in egg yolks. In this regard, it is feasible to produce n-3 PUFA (especially DHA)-enriched eggs, which are safe for adults and infants to consume by adding dietary rosehip meal as an antioxidant in laying hens’ diets. 

The increased content in n-3 PUFA and decrease in n-6 PUFA resulted in a lower n-6/n-3 ratio in yolks from hens fed RF1 and RF2 (Table 7). The nutritional quality of the eggs mostly related to the contents of SFA, MUFA, n-6, and n-3 PUFA, is currently of strong interest to reduce the ratio of n-6/n-3 PUFA in human diets. These quality indices are capable of reducing the risk of some chronic diseases, such as obesity, cardiovascular diseases, and certain forms of cancer, as well as improving brain development and function [45,46]. In our study, the n-6/n-3 ratio decreased significantly (*p* < 0.0001) in RF1 and RF2 yolks compared to RF0 yolks. The same effect was observed for the LA/ALA ratio. The peroxidability indices, which represent the susceptibility of lipid oxidation and the relationship between the FA, give us useful information in terms of shelf-life. This index also shows the relationship between PUFA content and antioxidant protection against the undesirable effects of oxidation [47]. Although we obtained higher values in RF1 and RF2 eggs compared to RF0, indicating a higher risk of autooxidation, the same eggs also have a higher healthy fat quality. However, rosehip meal was added as an antioxidant against lipid oxidation, and it was proven to be effective. From the nutritional quality indices group, the nutritive value was significantly (*p* = 0.0012) higher in RF1 and RF2 eggs, showing a good quality of the fat. The same effect was noted for desirable fatty acids (*p* = 0.0075). For human health, lower atherogenicity and thrombogenicity (less than 0.5 and 1 respectively), are recommended, due to their potential to stimulate platelet aggregation [6]. The different effects on cholesterol metabolism are given by the ratio between hypocholesterolemic and hypercholesterolemic fatty acids. Higher values are considered more beneficial for human health. Lastly, from the metabolic group of indices elongase was significantly higher (*p* = 0.0130) in the RF2 compared with RF0 while thioesterase was significant (*p* = 0.0037) in both RF1 and RF2 eggs. No effect was found for Δ9-desaturase (18) and Δ5/Δ6-desaturase. 

### 4.5. Effect of Rosehip and Flaxseed Meal on Antioxidant Activity

Natural antioxidants that are plant-derived products are not only characterized by being multifunctional and unharmful, but they have also been found to improve egg quality and the antioxidant capacity of laying hens’ eggs when incorporated into animal feed. As in the case of flaxseed meal, which is a natural source of fats, rosehip meal is a natural antioxidant source of plant origin containing bioactive compounds (polyphenols and antioxidant capacity) which can be transferred into the eggs. Thus, this study aimed to investigate whether the polyphenol content and antioxidant capacity of rosehip meal can increase the content of the same bioactive compounds in eggs. The results showed that rosehip meal exhibited a significant effect on TAC_a, TAC_y and TPC in eggs, showing a great potential to delay lipid and protein oxidation. The total antioxidant activity using the DPPH method of tested samples was between 48.91 mM Trolox (RF0) to 53.55 mM Trolox (RF2) in albumen samples and in yolk ranging from 50.09 (RF0) mM Trolox to 56.96 (RF2) mM Trolox. The highest concentration of TPC was observed in the RF2 eggs (46.79 μg GAE/g) compared to RF0 (40.93 μg GAE/g). Although to our knowledge there are no studies in which rosehip meal was used as a natural antioxidant for PUFA-enriched eggs, our results are comparable with studies that used different natural sources like rosemary [48,49], seabuckthorn, and grapeseed meal [6,50]. Studies in which flaxseed meals combined with synthetic antioxidants like vitamin E, butylated hydroxyanisole (BHA), and butylated hydroxytoluene (BHT) ([51,52] are available, however, the consumers’ concern regarding their safety has motivated the food industry to seek natural alternatives [53,54,55]. Therefore, the critical value of the application of natural antioxidants lies in their capacity to suppress oxidation processes, inhibit the free radical formation and/or interrupt propagation of autoxidation and reduce the levels of oxidative products in the animal and food system. Besides the diets, animal health status is also an important factor in the process of antioxidants’ deposition into the eggs [56,57]. It was previously reported that the antioxidative effects of rosehip were effective in defeating the reactive oxygen species due to substantial contributions from total polyphenols [58]. In this regard, antioxidant supplementation is necessary to maintain lipid and protein stability and protect PUFA during storage. However, further research is needed to investigate the different effects of rosehip in different conditions on lipid and protein stability during storage at different times and periods. 

### 4.6. Effect of Rosehip and Flaxseed Meal on Shelf-Life of Eggs

Eggs are food products that are highly perishable and their quality is lost when they are not properly handled and stored. Storage time and temperature are critical factors that affect the quality of eggs, due to physiochemical changes like weight loss, increased albumen pH, and flattening of the yolk [59], resulting in low-quality eggs. PUFA-enriched eggs are more susceptible to losing their quality due to the high content of fat, which is prone to early oxidation. Our results showed that by using rosehip meal as a source of antioxidant in PUFA-enriched eggs, the albumen and yolk quality, as well as the HU, were significantly higher than those from RF0 eggs. The effects of time and temperature were significant for almost all egg quality parameters during storage at 5 °C and 21 °C. However, the diet was noted to have a significant effect only on yolk pH, albumen pH, and Haugh unit. Modifications of the pH values in stored eggs are a normal process. During storage some chemical changes in egg components occur like carbon dioxide loss from the dissociation of carbonic acid, (forming water and carbon dioxide); breakdown of the protein structures of the albumen and vitelline membrane, which accelerates the passage of some components of the albumen passing through the yolk membrane [60]. Moreover, slowing the oxidative changes in stored eggs is crucial because the increase in pH during storage is attributed to the formation of ammonia and trimethylamine induced by autolytic reactions or microbial spoilage. Nevertheless, rosehip meal, delayed these processes, exhibited an antioxidant effect against lipid and protein oxidation in eggs during storage, and maintained their high quality, although some decline was noted in all eggs stored at 21 °C for 28 days. It has been shown that the decline in egg quality during storage is more rapid at higher temperatures compared to lower temperatures [61] which make the preservation of egg quality a critical interest for producers, consumers, and the food industry. This aspect is of critical value especially because the application of natural antioxidants lies in their capacity to suppress oxidation processes and reduce the levels of oxidative products in the animal and food system [62]. The weight loss in eggs from RF0 eggs after 28 days of storage at 5 °C was 4.82% and at 21 °C was 7.24%, whereas in RF1 and RF2 was only 2.35% and 1.13% respectively 3.92% and 3.65%. Egg weight loss influences the egg components and is a critical indicator for evaluating the freshness of the eggs, being associated with economic aspects [63]. The higher weight loss in eggs stored at 21 °C compared with those from refrigerated temperature was previously confirmed, and it happens due to the loss of moisture and gaseous products from the egg components [64,65]. Albumen pH was also significantly affected in RF0 compared with RF1 and RF2 eggs. After 28 days of storage, under the influence of the antioxidant capacity of rosehip, the values were maintained at optimum levels (between 7.5 to 8.5) whereas the RF0 albumen pH reached 9.66 at 21 °C. It was reported that the maximum level is 9.5 in commercial eggs [66,67]. These results reveal that albumen proteins are sensitive to storage time and temperature, which is imperative to preserve the protein albumen to extend the shelf life without the loss of its functional properties. At this point, the AA with antioxidant potential play an important role as main contributors to the antioxidant activity of the eggs. As it was reported, the antioxidant capacity is more sensitive to storage temperature and tends to decline during storage [68]. However, polyphenols are excellent antioxidants and shelf-life extenders due to their strong antioxidant effects and act as albumen preservers when deposited into the eggs. Further, the HU grade of eggs stored at 5 °C was found to be higher than that of eggs stored at 21 °C. However, both RF1 and RF2 eggs presented greater values than RF0. Generally, the higher HU value of stored eggs indicates better egg quality, being linked with albumen quality and showing better preservation, while the lower HU value of eggs indicates faster degradation [69], suggesting loss of albumen functional properties. Recently it was reported that when HU was lower than 70, albumen consistency was lost during storage [70]. Therefore, it is crucial to maintain higher HU values during storage to prevent protein and lipid peroxidation. Other studies [60,61,71,72,73], reported variable values (from 0 to 98.6) for HU and other egg quality parameters during storage (0 to 63 days) in different conditions (from 4 °C to 33 °C), however, none of them reported the effects of antioxidants on PUFA-enriched eggs. Literature data revealed that much attention has been given to the application of different feed additives to improve performance and, the quality of freshly laid eggs, showing that limited data are available on the effects of antioxidants in the diets of laying hens and on the preservation of egg quality during storage. Other natural antioxidants of plant origin, like green tea, rapeseed oil, acai lump, pumpkin seeds, and natural astaxanthin [22,71,74,75,76], were very effective in preserving the shelf-life of eggs, including albumen and yolk quality during storage at different times and temperatures. 

Nevertheless, there is still a need for studies regarding the effect of natural sources of antioxidants on PUFA-enriched eggs, to preserve their quality and shelf-life, while also taking into account the benefits provided by food industry wastes, which led to a minimum increase in production costs and obtaining high-quality products. 

## 5. Conclusions

The development of new products is a strategy that most of the food industries nowadays use to be competitive in the market. This approach leads to developing food products according to consumers’ wishes. The results of this study proved that the usage of rosehip meal in eggs enriched in polyunsaturated fatty acids exhibited a positive effect on amino acids and fatty acids content, and antioxidant capacity and was very effective in extending the shelf-life of eggs. Using dietary rosehip meal at a 3% inclusion level, in polyunsaturated fatty acids enriched eggs with 7% flaxseed meal, can be a natural way of designing functional food of animal origin, with a minimum increase in production costs.

## Figures and Tables

**Table 1:** Ingredients and nutritional composition of the diets.

Ingredients, % as Feed Basis	RF0	RF1	RF2
Corn	20.00	20.00	20.00
Wheat	28.25	24.47	24.18
Rice bran	10.00	10.00	10.00
Soybean meal	18.72	13.36	15.32
Rapeseed meal	8.00	8.00	4.68
Vegetable sunflower oil	3.53	3.84	3.9
Flaxseed meal	0.00	7.00	7.00
Rosehip meal	0.00	1.50	3.00
DL-Methionine	0.08	0.16	0.18
L-Lysine HCl	0.16	0.16	0.16
Calcium Carbonate	8.72	8.74	8.78
Monocalcium phosphate	1.27	1.33	1.36
Chloride	0.38	0.39	0.39
Choline	0.05	0.05	0.05
Premix vitamin–mineral	1.00	1.00	1.00
Total	100	100	100
Nutritional composition—Analyzed, %			
Dry matter	90.16	90.73	90.68
Crude protein	17.50	17.50	17.50
Crude fat	5.07	7.29	7.42
Crude fiber	3.99	5.64	5.97
Main fatty acid classes			
Saturated fatty acids (SFA)	18.45	15.99	15.24
Monounsaturated fatty acids (MUFA)	37.13	33.51	33.50
Polyunsaturated fatty acids (PUFA)	44.42	50.51	51.26
Unsaturated fatty acids (UFA)	81.55	84.04	84.76
n-3 PUFA	2.13	6.59	6.68
n-6 PUFA	42.29	43.92	44.58
n-6/n-3 ratio	19.88	6.67	6.68
Antioxidant capacity, mM Trolox	5.54	7.00	7.64

The premix contains: vitamin A:13.500 IU; vitamin D3:3.000 IU; vitamin E:27 mg; vitamin K3: 2 mg; vitamin B1: 2 mg; vitamin B2: 4.8 mg; pantothenic acid: 14.85 mg; nicotinic acid: 27 mg; vitamin B6: 3 mg; vitamin B7: 0.04 mg; vitamin B9:1 mg; vitamin B12: 0.018 mg; vitamin C: 25 mg; manganese: 71.9 mg; iron: 60 mg; copper: 6 mg; zinc: 60 mg; cobalt: 0.5 mg; iodine: 1.14 mg; selenium: 0.18 mg.

**Table 2:** Primary chemical composition, antioxidant compounds, fatty acids, and amino acids determined in rosehip meal and flaxseed meal.

Items	Rosehip Meal	Flaxseed Meal
Nutrients, %		
Dry matter	92.37	92.12
Crude protein	10.53	34.34
Crude fat	4.84	13.22
Crude fibre	49.35	12.51
Antioxidant compounds		
Total polyphenols, mg/g	60.23	nd
Antioxidant capacity, mM Trolox	23.87	nd
Flavonoids, mg Equiv. rutin/g	12.18	nd
Main fatty acids, (g/100 g FA)		
Palmitic C16:0	5.55	6.80
Stearic C18:0	2.98	3.20
Oleic cis C18:1	22.46	18.48
Linoleic cis C18:2n6	52.81	15.04
Linolenic α C18:3n3	14.28	55.36
PUFA	67.65	70.63
MUFA	22.80	18.75
SFA	9.55	10.43
n-3 PUFA	14.28	55.36
n-6 PUFA	53.07	15.27
n-6 to n-3 ratio	3.72	0.28
Amino acids, (g/100 g)		
Arginine	1.096	4.318
Isoleucine	0.401	1.681
Leucine	0.737	2.517
Lysine	0.242	1.497
Methionine	0.142	0.509
Phenylalanine	0.454	2.120
Threonine	0.419	1.849
Valine	0.444	1.970
EAA	3.935	15.953
Alanine	0.476	2.080
Aspartic acid	1.207	3.234
Glutamic acid	3.010	7.452
Serine	0.620	2.205
Glycine	0.671	2.174
Tyrosine	0.189	1.047
Cystine	0.204	0.649
NEAA	6.377	18.192
TAA	10.312	34.145

Nd: not determined; PUFA: polyunsaturated fatty acids; MUFA: monounsaturated fatty acids; SFA: saturated fatty acids; n-6 and n-3: omega; EAA: essential amino acids; NEAA: non-essential amino acids; TAA: total amino acids; n = 3.

**Table 3:** Effect of rosehip and flaxseed meals on laying hens’ performances.

Item	RF0	RF1	RF2	SEM	*p*
Daily feed intake, g feed/day	118.82 ^a^	116.41 ^b^	115.61 ^b^	0.129	0.0054
Feed conversion ratio, kg feed/kg egg	2.02	2.02	2.03	0.354	0.3214
Laying percentage, %	95.55 ^b^	96.67 ^ab^	98.38 ^a^	0.061	0.0013
Egg mass, g	64.60 ^b^	65.09 ^ab^	66.32 ^a^	0.021	0.0302

The significant differences (*p*  <  0.05) are marked in the tables by superscripts (a, b) after the 3 means were compared two by two, using the Fisher test. RF0: control diet; RF1: contain 1.5% rosehip meal and 7% flaxseed meal; RF2: contain 3% rosehip meal and 7% flaxseed meal; SEM: standard error of the mean; *p*: significance.

**Table 4:** Crude protein concentration and AA profile in fresh egg yolks.

Item (g/100 g)	RF0	RF1	RF2	SEM	*p*
Crude protein	31.44 ^b^	31.75 ^a^	31.80 ^a^	1.554	0.0450
Arginine	2.858 ^b^	2.986 ^a^	3.008 ^a^	0.927	0.0340
Isoleucine	1.691 ^b^	1.787 ^a^	1.796 ^a^	1.542	0.0315
Leucine	2.478 ^b^	2.689 ^a^	2.774 ^a^	0.024	0.0063
Lysine	2.384 ^b^	2.543 ^a^	2.549 ^a^	0.029	0.0075
Methionine	0.656 ^b^	0.722 ^a^	0.735 ^a^	0.845	0.0078
Phenylalanine	1.601 ^b^	1.905 ^a^	1.862 ^a^	1.224	0.0128
Threonine	2.348 ^b^	2.533 ^a^	2.521 ^a^	3.017	0.1440
Valine	1.505 ^b^	1.541 ^a^	1.557 ^a^	1.287	0.0030
EAA	15.521 ^b^	16.706 ^a^	16.802 ^a^	1.040	0.0432
Alanine	0.241 ^a^	0.191 ^b^	0.197 ^b^	0.010	0.0152
Aspartic acid	3.996 ^a^	3.614 ^b^	3.772 ^ab^	0.023	0.0081
Glutamic acid	4.602	4.347	4.352	1.108	0.1087
Serine	3.346 ^a^	3.192 ^a^	3.107 ^b^	0.209	0.0015
Glycine	0.831	0.847	0.851	0.715	0.0577
Tyrosine	1.712	1.621	1.653	0.334	0.2200
Cystine	0.835 ^b^	0.893 ^a^	0.911 ^a^	1.196	0.0319
NEAA	15.563 ^a^	14.705 ^b^	14.843 ^b^	2.557	0.0035
TAA	31.084 ^b^	31.411 ^a^	31.645 ^a^	0.222	0.0027

The significant differences (*p*  <  0.05) are marked in the tables by superscripts (a, b) after the 3 means were compared two by two, using the Fisher test. EAA: essential amino acids; NEAA: non-essential amino acids; TAA: total amino acids; RF0: control diet; RF1: contains 1.5% rosehip meal and 7% flaxseed meal; RF2: contains 3% rosehip meal and 7% flaxseed meal; SEM: standard error of the mean; *p*: significance.

**Table 5:** Effect of rosehip and flaxseed meals on egg yolk protein quality indices.

Item, %	RF0	RF1	RF2	SEM	*p*
TAAA	3.313 ^b^	3.526 ^a^	3.515 ^a^	0.073	0.0020
AAA	10.590 ^b^	10.596 ^ab^	10.768 ^a^	1.609	0.0065
SAA	1.491 ^b^	1.615 ^a^	1.646 ^a^	1.079	0.0146
TAAA/TAA	0.107 ^b^	0.112 ^a^	0.111 ^a^	0.089	0.0205
EAA/TAA	0.499 ^b^	0.532 ^a^	0.531 ^a^	0.015	0.0026
EAA/NEAA	0.997 ^b^	1.136 ^a^	1.132 ^a^	0.004	0.0268
Cystine/SAA	0.560 ^a^	0.553 ^b^	0.554 ^b^	0.055	0.0071

The significant differences (*p*  <  0.05) are marked in the tables by superscripts (a, b) after the 3 means were compared two by two, using the Fisher test. TAAA: total aromatic amino acids; AAA: amino acids with antioxidant potential; SAA: sulfur amino acids; EAA: essential amino acids; NEAA: non-essential amino acids; TAA: total amino acids; RF0: control diet; RF1: contain 1.5% rosehip meal and 7% flaxseed meal; RF2: contain 3% rosehip meal and 7% flaxseed meal; SEM: standard error of the mean; *p*: significance.

**Table 6:** Effect of rosehip and flaxseed meal on egg yolk fat and the fatty acid in fresh egg yolks.

Item (g/100 g)	RF0	RF1	RF2	SEM	*p*
Yolk fat	27.66	28.01	28.34	1.457	0.2295
Myristic C14:0	0.328 ^a^	0.263 ^b^	0.252 ^b^	0.022	0.0003
Pentadecanoic C15:0	0.072	0.077	0.075	0.008	0.4451
Palmitic C16:0	23.472 ^a^	21.968 ^b^	21.675 ^b^	0.882	0.0484
Heptadecanoic C17:0	0.157	0.117	0.117	0.064	0.3766
Stearic C18:0	11.583	11.415	12.297	1.846	0.7971
Total SFA	35.610	33.835	34.417	2.731	0.0825
Myristioleic C14:1	0.040 ^a^	0.025 ^b^	0.030 ^ab^	0.008	0.0224
Pentadecenoic C15:1	0.132	0.077	0.075	0.005	0.2569
Palmitoleic C16:1	23.472 ^a^	21.968 ^b^	21.675 ^b^	0.512	0.0404
Heptadecenoic C17:1	0.092	0.152	0.133	0.064	0.2049
Oleic C18:1	31.790 ^a^	30.645 ^b^	30.465 ^b^	0.512	0.0153
Erucic C22:1n9	0.083	0.078	0.070	0.041	0.6826
Nervonic C24:1n9	0.400 ^a^	0.230 ^b^	0.245 ^b^	0.021	<0.0001
Total MUFA	33.842	33.057	32.827	0.524	0.0591
Linoleic C18:2n6	22.810 ^b^	23.801 ^a^	23.182 ^ab^	1.120	0.0011
Linolenic γ C18:3n6	0.112	0.107	0.102	0.023	0.8217
Eicosadienoic C20:2n6	0.172	0.170	0.151	0.018	0.7489
Eicosatrienoic C20:3n6	0.332	0.298	0.292	0.045	0.2050
Arachidonic C20:4n6	4.457 ^a^	3.363 ^b^	3.887 ^b^	1.219	0.0088
Docosatetraenoic C22:4n6	1.790 ^a^	0.390 ^b^	0.355 ^b^	0.534	<0.0001
Total n-6	29.501 ^a^	28.192 ^ab^	27.969 ^b^	2.731	0.0312
Linolenic α C18:3n3	0.310 ^b^	1.382 ^a^	1.350 ^a^	0.103	<0.0001
Eicosatrienoic C20:3n3	0.219 ^b^	0.235 ^a^	0.281 ^a^	0.205	0.0298
Docosapentaenoic C22:5n3	0.084 ^b^	0.255 ^a^	0.288 ^a^	0.130	<0.0001
Docosahexaenoic C22:6n3	0.931 ^b^	2.695 ^a^	2.842 ^a^	0.076	<0.0001
Total n-3	1.544 ^b^	4.567 ^a^	4.761 ^a^	0.045	<0.0001
Total PUFA	31.045 ^b^	32.648 ^a^	32.730 ^a^	0.454	0.0033

The significant differences (*p*  <  0.05) are marked in the tables by superscripts (a, b) after the 3 means were compared two by two, using the Fisher test. PUFA: polyunsaturated fatty acids; MUFA: monounsaturated fatty acids; SFA: saturated fatty acids; n-3 and n-3: omega; RF0: control diet; RF1: contains 1.5% rosehip meal and 7% flaxseed meal; RF2: contains 3% rosehip meal and 7% flaxseed meal; SEM: standard error of the mean; *p*: significance.

**Table 7:** Estimation of lipid quality indices of egg yolk based on their nutritional classes.

Lipid Indices, %	RF0	RF1	RF2	SEM	*p*
Qualitative					
PUFA/SFA	0.862 ^b^	0.979 ^a^	0.952 ^a^	0.015	0.0009
n-6/n-3 FA	19.10 ^b^	6.17 ^b^	5.87 ^b^	1.565	<0.0001
Linoleic/α-Linolenic acids	70.85 ^a^	17.38 ^b^	18.01 ^b^	6.295	<0.0001
Peroxidability Index	65.36 ^b^	71.96 ^a^	71.98 ^a^	0.835	<0.0001
Nutritional					
Nutritional Value Index	1.81 ^b^	1.92 ^a^	1.97 ^a^	0.021	0.0012
Index of Atherogenicity	0.39 ^a^	0.35 ^b^	0.35 ^b^	0.006	0.0056
Index of Thrombogenicity	0.99 ^a^	0.76 ^b^	0.77 ^b^	0.030	<0.0001
Hypocholesterolemic/Hypercholesterolemic	2.59 ^b^	2.87 ^a^	2.88 ^a^	0.043	0.0017
Health-Promoting Index	2.60	2.87	2.89	0.043	0.0026
Desirable Fatty Acids	75.97 ^b^	77.58 ^a^	77.88 ^a^	0.263	0.0075
Metabolic					
Elongase Index	0.49 ^a^	0.52 ^ab^	0.56 ^b^	0.166	0.0130
Thioesterase Index	71.92 ^b^	84.16 ^a^	87.82 ^a^	5.140	0.0037
Δ9-Desaturase (18)	72.73	72.86	71.24	2.751	0.5558
Δ5/Δ6-Desaturase	21.15	21.14	22.49	0.075	0.2033

The significant differences (*p*  <  0.05) are marked in the tables by superscripts (a, b) after the 3 means were compared two by two, using the Fisher test. RF0: control diet; RF1: contains 1.5% rosehip meal and 7% flaxseed meal; RF2: contains 3% rosehip meal and 7% flaxseed meal; SEM: standard error of the mean; *p*: significance; Calculated on 20 samples per group.

**Table 8:** Influence of rosehip meal on shelf-life of eggs stored for 28 days at 5 °C and 21 °C.

Item		External Quality Parameters				Internal Quality Parameters			Shell Quality	
		Egg, g	Albumen, g	Yolk, g	Shell, g	Yolk pH	Albumen pH	HU	Thick Nesses	Breaking Strength
0 d	RF0	64.23	39.67	16.54	8.02	5.72	8.11	82.70	0.36	3.99
	RF1	64.48	39.80	15.96	7.72	5.71	8.09	94.13	0.31	4.14
	RF2	64.37	40.31	16.57	7.48	5.73	8.09	93.83	0.34	4.84
14 d	5 °C									
	RF0	63.33	39.05	15.65	7.97	6.08	8.92	88.18	0.34	3.90
	RF1	63.94	39.26	15.85	7.49	6.09	8.88	91.50	0.37	4.15
	RF2	64.08	40.68	16.32	7.86	6.08	8.81	91.13	0.37	4.64
	21 °C									
	RF0	62.67	38.14	16.76	7.80	6.26	9.09	79.94	0.35	3.91
	RF1	62.88	37.25	16.94	7.49	6.23	8.83	81.45	0.36	3.90
	RF2	63.04	37.76	17.20	7.44	6.20	8.74	81.69	0.37	4.23
28 d	5 °C									
	RF0	61.13	38.08	16.48	7.61	6.28	9.01	86.32	0.35	4.25
	RF1	62.96	37.89	16.89	7.70	6.22	8.76	89.01	0.36	4.11
	RF2	63.64	38.09	16.89	7.66	6.15	8.61	90.17	0.34	4.15
	21 °C									
	RF0	59.58	33.79	18.12	7.68	6.44	9.66	72.95	0.34	3.58
	RF1	61.95	33.33	17.39	7.51	6.35	9.04	78.45	0.34	4.20
	RF2	62.02	35.35	17.04	7.63	6.30	9.01	79.71	0.34	4.18
SEM		0.203	0.193	0.122	0.047	0.007	0.007	0.485	0.003	0.061
**Main effect**										
***Time***										
0 d		64.36^a^	39.93 ^a^	16.36 ^b^	7.74	5.72 ^c^	8.09 ^c^	90.22 ^a^	0.34 b	4.32
14 d		63.01^b^	38.69 ^a^	16.45 ^b^	7.68	6.16 ^b^	8.98 ^a^	85.65 ^b^	0.36 a	4.12
28 d		60.88^c^	36.09 ^a^	17.14 ^a^	7.63	6.29 ^a^	8.90 ^b^	82.27 ^c^	0.34 b	4.09
***Temperature***										
5 °C		63.12^a^	38.84 ^a^	16.35 ^b^	7.71	6.15 ^b^	8.85 ^b^	89.22 ^a^	0.36	4.12
21 °C		60.77^b^	35.94 ^b^	17.24 ^a^	7.59	6.30 ^a^	9.03 ^a^	78.70 ^b^	0.35	4.00
***Diet***										
RF0		62.43	37.74	16.84	7.58 ^b^	6.16 ^a^	8.82 ^a^	81.02 ^b^	0.35	3.92b
RF1		62.18	37.79	16.61	7.61 ^a^	6.12 ^ab^	8.76 ^b^	85.51 ^b^	0.35	4.10b
RF2		62.67	38.15	16.67	7.82 ^a^	6.09 ^b^	8.73 ^b^	87.11 ^a^	0.35	4.41a
***p***										
time		<0.001	<0.001	0.014	0.685	<0.001	<0.001	0.003	0.023	0.763
temp		<0.001	<0.001	0.002	0.251	<0.001	<0.001	<0.001	0.528	0.149
diet		0.713	0.625	0.668	0.066	0.013	<0.001	<0.001	0.662	0.003
time × temp		0.067	0.029	0.625	0.480	0.960	0.302	0.243	0.567	0.902
time × diet		0.830	0.341	0.936	0.370	0.031	0.074	0.637	0.336	0.479
temp × diet		0.322	0.187	0.929	0.763	0.553	<0.001	0.982	0.510	0.753
time × temp × diet		0.894	0.489	0.145	0.499	0.650	0.704	0.677	0.823	0.185

The significant differences (*p*  <  0.05) are marked in the tables by superscripts (a, b, c) after the 3 means were compared two by two, using the Fisher test. RF0: control diet; RF1: contains 1.5% rosehip meal and 7% flaxseed meal; RF2: contains 3% rosehip meal and 7% flaxseed meal; SEM: standard error of the mean; *p*: significance; d: days of storage.

## Data Availability

The data presented in this study are available in the article.

## References

[1] Lorenzo J.M., Munekata P.E., Pateiro M., Fierro E.M., Brnčić S.R., Brnčić M., Barba F.J. (2020). Functional foods. Nutraceuticals and Dietary Supplements.

[2] Birch C.S., Bonwick G.A. (2019). Ensuring the future of functional foods. Int. J. Food Sci. Technol..

[3] Topolska K., Florkiewicz A., Filipiak-Florkiewicz A. (2021). Functional Food—Consumer Motivations and Expectations. Int. J. Environ. Res. Public Health.

[4] Wu Y.B., Li L., Wen Z.G., Yan H.J., Yang P.L., Tang J., Xie M., Hou S.S. (2019). Dual functions of eicosapentaenoic acid-rich microalgae: Enrichment of yolk with n-3 polyunsaturated fatty acids and partial replacement for soybean meal in diet of laying hens. Poult. Sci..

[5] Oomah B.D. (2020). Flaxseed By-products. Food Wastes and By-Products: Nutraceutical and Health Potential.

[6] Vlaicu P.A., Panaite T.D., Turcu R.P. (2021). Enriching laying hens eggs by feeding diets with different fatty acid composition and antioxidants. Sci. Rep..

[7] Shayan M., Kamalian S., Sahebkar A., Tayarani-Najaran Z. (2020). Flaxseed for Health and Disease: Review of Clinical Trials. CCHTS.

[8] Igual M., Chiş M.S., Păucean A., Vodnar D.C., Muste S., Man S., Martínez-Monzó J., García-Segovia P. (2021). Valorization of Rose Hip (*Rosa canina*) Puree Co-Product in Enriched Corn Extrudates. Foods.

[9] Mármol I., Sánchez-de-Diego C., Jiménez-Moreno N., Ancín-Azpilicueta C., Rodríguez-Yoldi M.J. (2017). Therapeutic Applications of Rose Hips from Different Rosa Species. Int. J. Mol. Sci..

[10] Medveckienė B., Kulaitienė J., Levickienė D., Hallmann E. (2021). The Effect of Ripening Stages on the Accumulation of Carotenoids, Polyphenols and Vitamin C in Rosehip Species/Cultivars. Appl. Sci..

[11] Kubczak M., Khassenova A.B., Skalski B., Michlewska S., Wielanek M., Aralbayeva A.N., Murzakhmetova M.K., Zamaraeva M., Skłodowska M., Bryszewska M. (2020). Bioactive Compounds and Antiradical Activity of the *Rosa canina* L. Leaf and Twig Extracts. Agronomy.

[12] Patel S. (2017). Rose hip as an underutilized functional food: Evidence-based review. Trends Food Sci. Technol..

[13] Gjorgovska N., Grigorova S., Levkov V. (2021). Application of Rose Hip Fruits as Feed Supplement in Animal Nutrition. J. Agric. Food Dev..

[14] Konca Y., Kaliber M., Uzkulekci H.H., Cimen B., Yalcin H. (2021). The effect of rosehip (*Rosa canina* L.) supplementation to diet on the performance, egg and meat quality, antioxidant activity in laying quail. Sains Malays..

[15] Olteanu M., Panaite T.D., Turcu R.P., Ropota M., Vlaicu P.A., Mitoi M. (2022). Using grapeseed meal as natural antioxidant in slow-growing Hubbard broiler diets enriched in polyunsaturated fatty acids. Rev. Mex. Cienc. Pecu..

[16] Varzaru I., Untea A.E., Martura T., Olteanu M., Panaite T.D., Schitea M., Van I. (2013). Development and validation of an RP-HPLC method for methionine, cystine and lysine separation and determination in corn samples. Rev. Chem..

[17] Food and Agriculture Organization (FAO), World Health Organization (WHO), United Nations University (UNU) (2007). Protein and Amino Acid Requirements in Human Nutrition.

[18] Turcu R.P., Olteanu M., Criste R.D., Panaite T.D., Ropotă M., Vlaicu P.A., Drăgotoiu D. (2019). Grapeseed meal used as natural antioxidant in high fatty acid diets for Hubbard broilers. Braz. J. Poult. Sci..

[19] Untea A.E., Varzaru I., Panaite T.D., Gavris T., Lupu A., Ropota M. (2020). The Effects of dietary inclusion of bilberry and walnut leaves in laying hens’ diets on the antioxidant properties of eggs. Animals.

[20] FAO/WHO (Food and Agricultural Organization of the United Nations and World Health Organization) (2010). Fats and Fatty Acids in Human Nutrition.

[21] Olteanu M., Criste R.D., Panaite T.D., Ropotă M., Mitoi M., Vlaicu P.A., Șoica C. (2017). Quality of the eggs obtained from hens fed diet formulations rich in polyunsaturated fatty acids and with grape seeds meal as antioxidant. Arch. Zootech..

[22] Vlaicu P.A., Panaite T.D. (2022). Effect of dietary pumpkin (*Cucurbita moschata*) seed meal on layer performance and egg quality characteristics. Anim. Biosci..

[23] Zimniewska M., Rozańska W., Gryszczynska A., Romanowska B., Kicinska-Jakubowska A. (2018). Antioxidant Potential of Hemp and Flax Fibers Depending on Their Chemical Composition. Molecules.

[24] Lemes L.F.R., Tarley C.R.T. (2021). Combination of supramolecular solvent-based microextraction and ultrasound-assisted extraction for cadmium determination in flaxseed flour by thermospray flame furnace atomic absorption spectrometry. Food Chem..

[25] Moldovan C., Babotă M., Mocan A., Menghini L., Cesa S., Gavan A., Sisea C., Vodnar D.C., Dias M.I., Pereira C. (2021). Optimization of the drying process of autumn fruits rich in antioxidants: A study focusing on rosehip (*Rosa canina* L.) and sea buckthorn (*Elaeagnus rhamnoides* (L.) A. Nelson) and their bioactive properties. Food Funct..

[26] Grigorova S., Gjorgovska N., Levkov V. (2021). Effects of rosehip feed supplementation on egg quality parameters, yolk lipid oxidation, and blood parameters of laying hens. Iran. J. Appl. Anim. Sci..

[27] Sepehr A., Kashani R.B., Esmaeili M., Safari O., Rombenso A. (2021). Effects of extruded, milled, and whole flaxseed (*Linum usitatissimum*) on egg performance, lipid components, and fatty acids concentrations in yolk and blood, and antioxidant system of commercial laying hens. Anim. Feed. Sci. Technol..

[28] Huang S., Baurhoo B., Mustafa A. (2018). Effects of extruded flaxseed on layer performance, nutrient retention and yolk fatty acid composition. Br. Poult. Sci..

[29] Bean L.D., Leeson S. (2003). Long-term effects of feeding flaxseed on performance and egg fatty acid composition of brown and white hens. Poult. Sci..

[30] Imran M., Anjum F.M., Nadeem M., Ahmad N., Khan M.K., Mushtaq Z., Hussain S. (2015). Production of Bio-omega-3 eggs through the supplementation of extruded flaxseed meal in hen diet. Lipids Health Dis..

[31] Hatice K., Adem K., Esenbuğa N., Macit M. (2019). The effect of rosehip seed supplementation into laying hens diets on performance, egg quality traits, yolk lipid profile and serum parameters. Alinteri J. Agric. Sci..

[32] Bortoluzzi C., Rochell S.J., Applegate T.J. (2018). Threonine, arginine, and glutamine: Influences on intestinal physiology, immunology, and microbiology in broilers. Poult. Sci..

[33] Debnath B.C., Biswas P., Roy B. (2019). The effects of supplemental threonine on performance, carcass characteristics, immune response and gut health of broilers in subtropics during pre-starter and starter period. J. Anim. Phy. Anim. Nutr..

[34] Ravindran V. (2013). Poultry feed availability and nutrition in developing countries. Poult. Dev. Rev..

[35] Heger J., Frydrych Z. (2019). Efficiency of utilization of amino acids. Absorption and Utilization of Amino Acids.

[36] Attia Y.A., Al-Harthi M.A., Korish M.A., Shiboob M.H. (2020). Protein and Amino Acid Content in Four Brands of Commercial Table Eggs in Retail Markets in Relation to Human Requirements. Animals.

[37] Nishimura K., Ijiri D., Shimamoto S., Takaya M., Ohtsuka A., Goto T. (2021). Genetic effect on free amino acid contents of egg yolk and albumen using five different chicken genotypes under floor rearing system. PLoS ONE.

[38] Tas N.G., Gokmen V. (2018). Profiling of the contents of amino acids, water-soluble vitamins, minerals, sugars and organic acids in Turkish hazelnut varieties. Pol. J. Food Nutr. Sci..

[39] Jensen C.L., Maude M., Anderson R.E., Heird W.C. (2000). Effect of docosahexaenoic acid supplementation of lactating women on the fatty acid composition of breast milk lipids and maternal and infant plasma phospholipids. Am. J. Clin. Nutr..

[40] Vlaicu P.A., Untea A.E., Turcu R.P., Saracila M., Panaite T.D., Cornescu G.M. (2022). Nutritional composition and bioactive compounds of basil, thyme and sage plant additives and their functionality on broiler thigh meat quality. Foods.

[41] Fredriksson S., Elwinger K., Pickova J. (2000). Fatty acid and carotenoid composition of egg yolk as an effect of microalgae addition to feed formula for laying hens. Food Chem..

[42] Fraeye I., Bruneel C., Lemahieu C., Buyse J., Muylaert K., Foubert I. (2012). Dietary enrichment of eggs with omega-3 fatty acids: A review. Food Res. Int..

[43] Gładkowski W., Kiełbowicz G., Chojnacka A., Bobak Ł., Spychaj R., Dobrzański Z., Trziszka T., Wawrzeńczyk C. (2014). The effect of feed supplementation with dietary sources of n-3 polyunsaturated fatty acids, flaxseed and algae *Schizochytrium* sp., on their incorporation into lipid fractions of J apanese quail eggs. Int. J. Food Sci. Technol..

[44] Lemahieu C., Bruneel C., Termote-Verhalle R., Muylaert K., Buyse J., Foubert I. (2014). Effect of different microalgal n− 3 PUFA supplementation doses on yolk color and n− 3 LC-PUFA enrichment in the egg. Algal Res..

[45] Pottel L., Lycke M., Boterberg T., Foubert I., Pottel H., Duprez F., Goethals L., Debruyne P.R. (2014). Omega-3 fatty acids: Physiology, biological sources and potential applications in supportive cancer care. Phytochem. Rev..

[46] Gogus U., Smith C. (2010). n-3 Omega fatty acids: A review of current knowledge. Int. J. Food Sci. Technol..

[47] Kang M.J., Shin M.S., Park J.N., Lee S.S. (2005). The effects of polyunsaturated: Saturated fatty acids ratios and peroxidisability index values of dietary fats on serum lipid profiles and hepatic enzyme activities in rats. Br. J. Nutr..

[48] Galobart J., Barroeta A.C., Baucells M.D., Codony R., Ternes W. (2001). Effect of dietary supplementation with rosemary extract and α-tocopheryl acetate on lipid oxidation in eggs enriched with ω3-fatty acids. Poult. Sci..

[49] Harlina P.W., Ma M., Shahzad R., Khalifa I. (2022). Effect of Rosemary Extract on Lipid Oxidation, Fatty Acid Composition, Antioxidant Capacity, and Volatile Compounds of Salted Duck Eggs. Food Sci. Anim. Resour..

[50] Multescu M., Marinas I.C., Susman I.E., Belc N. (2022). Byproducts (Flour, Meals, and Groats) from the Vegetable Oil Industry as a Potential Source of Antioxidants. Foods.

[51] Hayat Z., Cherian G., Pasha T.N., Khattak F.M., Jabbar M.A. (2009). Effect of feeding flax and two types of antioxidants on egg production, egg quality, and lipid composition of eggs. J. Appl. Poult. Res..

[52] Hayat Z., Cherian G., Pasha T.N., Khattak F.M., Jabbar M.A. (2010). Oxidative stability and lipid components of eggs from flax-fed hens: Effect of dietary antioxidants and storage. Poult. Sci..

[53] Brewer M.S. (2011). Natural antioxidants: Sources, compounds, mechanisms of action, and potential applications. Compr. Rev. Food Sci. Food Saf..

[54] Chiang Y.-F., Chen H.-Y., Ali M., Shieh T.-M., Huang Y.-J., Wang K.-L., Chang H.-Y., Huang T.-C., Hong Y.-H., Hsia S.-M. (2021). The Role of Cell Proliferation and Extracellular Matrix Accumulation Induced by Food Additive Butylated Hydroxytoluene in Uterine Leiomyoma. Nutrients.

[55] Xu X., Liu A., Hu S., Ares I., Martínez-Larrañaga M.R., Wang X., Martínez M., Anadón A., Martínez M.A. (2021). Synthetic phenolic antioxidants: Metabolism, hazards and mechanism of action. Food Chem..

[56] Curpan A.S., Impellitteri F., Plavan G., Ciobica A., Faggio C. (2022). Mytilus galloprovincialis: An essential, low-cost model organism for the impact of xenobiotics on oxidative stress and public health. Comp. Biochem. Physiol. Part C Toxicol. Pharmacol..

[57] Soldado D., Bessa R.J.B., Jerónimo E. (2021). Condensed Tannins as Antioxidants in Ruminants—Effectiveness and Action Mechanisms to Improve Animal Antioxidant Status and Oxidative Stability of Products. Animals.

[58] Daels-Rakotoarison D.A., Gressier B., Trotin F., Brunet C., Luyckx M., Dine T., Bailleul F., Cazin M., Cazin J.C. (2002). Effects of *Rosa canina* fruit extract on neutrophil respiratory burst. Phytother. Res. Int. J. Devoted Pharmacol. Toxicol. Eval. Nat. Prod. Deriv..

[59] Yuceer M., Caner C. (2014). Antimicrobial lysozyme–chitosan coatings affect functional properties and shelf life of chicken eggs during storage. J. Sci. Food Agric..

[60] Vlaicu P.A., Panaite T.D., Cornescu G.M. (2021). Shelf life of eggs from hens fed diets rich in polyunsaturated fatty acids and antioxidants under the effect of different storage time and temperatures. Sci. Pap. Ser. D Anim. Sci..

[61] Feddern V., de Prá M.C., Mores R., Nicoloso R.d.S., Coldebella A., Abreu P.G.d. (2017). Egg quality assessment at different storage conditions, seasons and laying hen strains. Ciênc. Agrotecnol..

[62] Elwan H.A., Elnesr S.S., Mohany M., Al-Rejaie S.S. (2019). The effects of dietary tomato powder (*Solanum lycopersicum* L.) supplementation on the haematological, immunological, serum biochemical and antioxidant parameters of growing rabbits. J. Anim. Physiol. Anim. Nutr..

[63] Pires P.G.D.S., Leuven A.F.R., Franceschi C.H., Machado G.S., Pires P.D.D.S., Moraes P.D.O., Kindlein L., Andretta I. (2020). Effects of rice protein coating enriched with essential oils on internal quality and shelf life of eggs during room temperature storage. Poult. Sci..

[64] Eke M.O., Olaitan N.I., Ochefu J.H. (2013). Effect of storage conditions on the quality attributes of shell (table) eggs. Niger. Food J..

[65] Sati N.M., Oshibanjo D.O., Emennaa P.E., Mbuka J.J., Haliru H., Ponfa S.B., Abimiku O.R., Nwamo A.C. (2020). Egg Quality Assessment within Day 0 to 10 as Affected by Storage Temperature. Asian J. Res. Anim. Vet. Sci..

[66] Adamski M., Kuzniacka J., Czarnecki R., Kucharska-Gaca J., Kowalska E. (2017). Variation in egg quality traits depending on storage conditions. Pol. J. Natl. Sci..

[67] Marzec A., Damaziak K., Kowalska H., Riedel J., Michalczuk M., Koczywąs E., Cisneros F., Lenart A., Niemiec J. (2019). Effect of Hens Age and Storage Time on Functional and Physiochemical Properties of Eggs. J. Appl. Poult. Res..

[68] Nimalaratne C., Schieber A., Wu J. (2016). Effects of storage and cooking on the antioxidant capacity of laying hen eggs. Food Chem..

[69] Jones D.R., Ward G.E., Regmi P., Karcher D.M. (2018). Impact of egg handling and conditions during extended storage on egg quality. Poult. Sci..

[70] De Araújo Soares R., Borges S.V., Dias M.V., Piccoli R.H., Fassani E.J., da Silva E.M.C. (2021). Impact of whey protein isolate/sodium montmorillonite/sodium metabisulfite coating on the shelf life of fresh eggs during storage. LWT.

[71] Heng N., Gao S., Guo Y., Chen Y., Wang L., Sheng X., Wang X., Xing K., Xiao L., Ni H. (2020). Effects of supplementing natural astaxanthin from Haematococcus pluvialis to laying hens on egg quality during storage at 4 °C and 25 °C. Poult. Sci..

[72] Tabib I., Onbaşilar E.E., Yalçin S. (2021). The effects of cage type, oviposition time and egg storage period on the egg quality characteristics of laying hens. Ankara Univ. Vet. Fak. Derg..

[73] Lee J., Seo H.G., Lee C.-H. (2020). Effects of lotus (*Nelumbo nucifera*) leaf hot water extracts on the quality and stability of eggs using ultrasonication treatment during storage. Food Sci. Anim. Res..

[74] Yuan N., Wang J.P., Ding X.M., Bai S.P., Zeng Q.F., Su Z.W., Xuan Y., Peng H.W., Fraley G.S., Zhang K.Y. (2019). Effects of supplementation with different rapeseed oil sources and levels on production performance, egg quality, and serum parameters in laying hens. Poult. Sci..

[75] Zhang J., Zhang M., Liang W., Geng Z., Chen X. (2020). Green tea powder supplementation increased viscosity and decreased lysozyme activity of egg white during storage of eggs frjmom Huainan partridge chicken. Ital. J. Anim. Sci..

[76] Fortuoso B.F., Gebert R.R., De Oliveira R.C., Boiago M.M., Souza C.F., Baldissera M.D., Vendruscolo R.G., Kempka A.P., Paiano D., Wagner R. (2019). Impacts of the supplementation of acai lump flour in the diet of laying hens on productive performance, and fatty acid profiles and antioxidant capacity in the fresh and stocked eggs. J. Food Biochem..

